# Post-marketing active surveillance of Guillain Barré Syndrome following COVID-19 vaccination in persons aged ≥12 years in Italy: A multi-database self-controlled case series study

**DOI:** 10.1371/journal.pone.0290879

**Published:** 2024-01-19

**Authors:** Cristina Morciano, Stefania Spila Alegiani, Francesca Menniti Ippolito, Valeria Belleudi, Gianluca Trifirò, Giovanna Zanoni, Aurora Puccini, Ester Sapigni, Nadia Mores, Olivia Leoni, Giuseppe Monaco, Elena Clagnan, Cristina Zappetti, Emanuela Bovo, Maria Cutillo, Roberto Da Cas, Marco Massari

**Affiliations:** 1 Pharmacoepidemiology and Pharmacosurveillance Unit, National Centre for Drug Research and Evaluation, Istituto Superiore di Sanità (National Institute of Health), Rome, Italy; 2 Department of Epidemiology ASL Roma 1, Lazio Regional Health Service, Rome, Italy; 3 Department of Diagnostics and Public Health, University of Verona, Verona, Italy; 4 Immunology Unit, University Hospital, Verona, Italy; 5 Hospital Assistance Service, General Directorate for Personal Care, Health and Welfare, Emilia-Romagna Region, Bologna, Italy; 6 Institute of Pharmacology, Pharmacovigilance, Policlinico Universitario A. Gemelli, Catholic University of Sacred Heart, Rome, Italy; 7 Department of Health of Lombardy Region, Epidemiology Observatory, Milan, Italy; 8 Regional Health Authority, Friuli Venezia Giulia Region, Udine, Italy; 9 Central Directorate for Health, Social Policies, Friuli Venezia Giulia Region, Trieste, Italy; 10 Veneto Tumour Registry, Azienda Zero, Padova, Italy; University of Bologna / Romagna Local Health Authority, ITALY

## Abstract

**Background:**

Recently published studies have reported association of COVID-19 vaccine ChAdOx1-S (Vaxzevria) with Guillain Barré Syndrome (GBS). Less is known about the safety of other COVID-19 vaccines with respect to GBS outcome. This study investigated the association of COVID-19 vaccines with GBS in more than 15 million persons aged ≥12 years in Italy.

**Methods:**

Study population was all individuals aged ≥12 years who received at least one dose of COVID-19 vaccines, admitted to emergency care/hospital for GBS from 27 December 2020–30 September 2021 in Italy. Identification of GBS cases and receipt of at least one dose of mRNA-1273 (Elasomeran), BNT162b2 (Tozinameran), ChAdOx1-S (Vaxzevria) and Ad26.COV2.S (Janssen) through record linkage between regional health care and vaccination registries. Relative Incidence (RI) was estimated Self-controlled case series method adapted to event-dependent exposure using in the 42-day exposure risk period after each dose compared with other observation periods.

**Results:**

Increased risk of GBS was found after first (RI = 6.83; 95% CI 2.14–21.85) and second dose (RI = 7.41; 2.35–23.38) of mRNA-1273 and first dose of ChAdOx1-S (RI = 6.52; 2.88–14.77). Analysis by age found an increased risk among those aged≥60 years after first (RI = 8.03; 2.08–31.03) and second dose (RI = 7.71; 2.38–24.97) of mRNA-1273. The first dose of ChAdOx1-S was associated with GBS in those aged 40–59 (RI = 4.50; 1.37–14.79) and in those aged ≥ 60 years (RI = 6.84; 2.56–18.28).

**Conclusions:**

mRNA-1273 and ChAdOx1-S vaccines were associated with an increased risk of GBS however this risk resulted in a small number of excess cases. Limitations were loss of GBS outpatient cases and imprecision of the estimates in the subgroup analysis due to a low number of events.

## Introduction

There have been reports of cases of Guillain-Barré (GBS) after mRNA [1, 2] and adenovirus-vectored COVID-19 vaccination [3, 4]. The European Medicine Agency has listed GBS as a rare side effect related to Chadox1-S (marked in EU as Vaxzevria) and Ad26.COV2-S (marked in EU as Janssen-Jcovden) [5, 6]. In United States, the Food and Drug Administration issued a warning on the increased risk of GBS following Ad26.COV2-S- vaccine [7]. In UK, the Medicines and Healthcare products Regulatory Agency updated product information for Chadox1-S (marked in UK as AstraZeneca) to include GBS in the adverse reactions associated with the vaccine [8].

To date, post-marketing analytical studies have been conducted to examine the potential association of GBS with COVID-19 vaccines. A self-controlled case series (SCCS) study in England [9] reported an increased risk of GBS with Chadox1 but not with BNT162b2. Another SCCS study in England [10] found an association of GBS with Chadox1 and no association with BNT162b2 nor with mRNA-1273. However, due to the limited availability of vaccine data at that time, these two England-based studies were able to provide a partial safety profile of the COVID-19 vaccines with respect to GBS, with one [9] limiting the analysis to BNT162b2 and Chadox1 vaccines (first dose) and the other examining the effect of only one dose of mRNA-1273 vaccine [10]. In United States, a cohort study found an association of GBS with Ad.26.COV2.S but not with mRNA vaccines combined [11].

In Italy, vaccination campaign started on 27 December 2020 and four vaccines have been authorized and widely used. These include two mRNA vaccines, BNT162b2 (Comirnaty-Tozinameran) and mRNA-1273 (Moderna-Elasomeran) and two adenovirus-vectored vaccines, ChAdOx1-S and Ad26.COV2-S.

The National Institute of Health (Istituto Superiore di Sanità) and the Italian Medicines Agency (Agenzia Italiana del Farmaco) coordinate a national active post-marketing surveillance on effectiveness and safety of COVID-19 vaccines in Italy [12]. The active surveillance is based on record linkage among several regional health care databases and vaccination registries, using TheShinISS, an R-based open-source statistical tool, developed by the National Institute of Health [13].

As part of this surveillance, we conducted a SCCS study to evaluate the risk of GBS after vaccination with BNT162b2, mRNA-1273, ChAdOx1-S and Ad26.COV2-S in the population aged 12 years or older, based on data from 27 December 2020 to 30 September 2021.

## Methods

### Data source

The active surveillance is based on a dynamic multi-regional observational cohort. A distributed analysis framework is applied using TheShinISS, an R-based open-source statistical tool, developed by the researchers of the National Institute of Health [13], that locally processes data collected and updated periodically from regional health care databases according to ad hoc, study-tailored, common data model.

Data sources for the identification and characterization of GBS cases have a high level of coverage and accuracy because their collection is mandatory by law in order to obtain the reimbursement within the Italian National Health Service (NHS). Data on vaccination exposure, on emergency care/hospital admission for GBS and subjects’ characteristics were retrieved from several routinely collected regional healthcare databases:

- COVID-19 vaccination registry to identify information on administered vaccines (product, date of administration and doses for all vaccinated subjects);
- population registry to identify information on age, sex and vital status (causes of death are not recorded in this registry);
- hospital discharge and emergency care visit databases to identify GBS events in the period pre- and post-vaccination, and information on the comorbidities of the study subjects in the period preceding the vaccination, codified according to the 9^th^ International Classification of Diseases (ICD-9-CM);
- pharmacy claims and copayment exemptions databases to obtain information on the comorbidities of the study subjects in the period preceding the vaccination;
- vaccination registry to identify vaccinations other than anti-COVID-19 (e.g., flu and pneumococcal vaccines) administered in the period pre- and post-anti COVID-19 vaccination;
- COVID-19 surveillance system to obtain information on SARS-CoV2 infection and related outcomes.

Regional health data were locally transformed into a study-specific Common Data Model and locally processed using *TheShinISS*. Quality check was executed both at local and central level (National Institute of Health) using TheShinISS tool. Authors did not have access to information that could identify individual participants during or after data collection.

**Fig 1:**
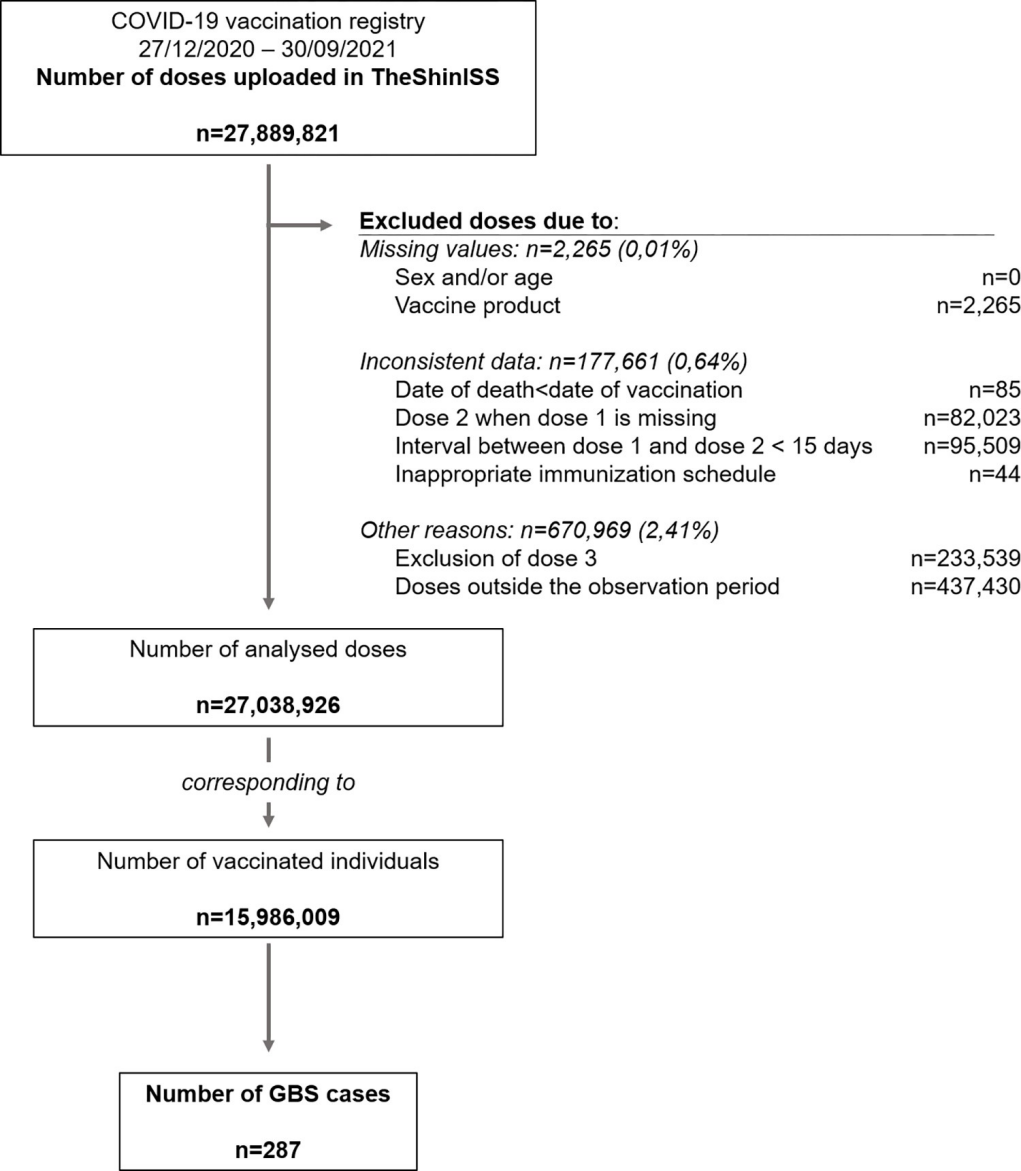
Flow chart of selection of the study population (27 December 2020–30 September 2021).

In the end, regional pseudonymized datasets were provided to the National Institute of Health for centralized analysis, in compliance with EU General Data Protection Regulation. Over the last two years, *TheShinISS* framework has been employed in several large-scale observational studies exploring the association between some exposures and COVID-19 onset/prognosis as well as other drug and vaccine-related research topics and is currently maintained by a collaborative research network [12–18]. The relational scheme of the study databases as well as TheShinISS flow diagram is described in Fig 1 in the article by Massari et al [12].

### Study period and population

We investigated the association between anti-COVID-19 vaccines and subsequent onset of GBS in the population aged ≥12 years. Five Italian Regions (northern Italy: Lombardia, Veneto, Friuli Venezia Giulia and Emilia Romagna; central Italy: Lazio), representing 44% of the population aged ≥12 years resident in Italy, contributed data of all vaccinated persons in this age group, in a period ranging from 27/12/2020 to 30 September 2021, based on the last data update, which varied across Regions: Lombardia up to 30/09/2021, Veneto up to 20/06/2021, Friuli Venezia Giulia up to 31/08/2021, Emilia Romagna up to 30/06/2021 and Lazio up to 16/06/2021. We included in the study all persons aged ≥12 years who received at least a first dose of anti-COVID-19 vaccines and were admitted to emergency care or hospital with the outcome of GBS. We excluded individuals with missing or inconsistent information on relevant variables (age, sex, vaccine product and dose, date of vaccination, of death and of event) as well as individuals with a history of GBS within 365 days prior to the start of the study period.

The observation period for each case ranged from 27 December 2020 to the end of follow-up, which occurred at the end of Region-specific study period. If patients died, the end of the observation period was defined according to what is proposed by the SCCS methodology to handle mortality [19].

### Study design

We used a SCCS study design [20–24]. The SCCS study design has emerged as a key methodology for studying the safety of vaccines. This approach requires information only from individuals who have experienced the event of interest (cases). Individuals act as their own control and estimation is performed within individuals during the study period. It follows that this design allows to automatically control for multiplicative time-invariant confounders, even when these are unmeasured or unknown. Originally designed to analyze the association between vaccination and specific events under the key assumption that events do not influence post-event exposures, this method has been adapted to event-dependent exposures, for example when occurrence of an event may preclude any subsequent exposure (SCCS method for censored, perturbed or curtailed post-event exposures) [19, 23, 24]. This is the case in observational studies of vaccines when the event of interest could be a contraindication to vaccination.

By using the adapted SCCS method for event-dependent exposures, we estimated the Relative Incidence (RI) of GBS following pre-specified windows at risk after vaccination, in a within-person comparison of different time-periods. The method allows for the control of all time-independent characteristics of subjects. The SCCS method allows also for adjustment of potential time-varying confounders such as seasonal variation in risks.

### Definition of outcomes

The outcome of interest was the first diagnosis of GBS identified from emergency care and/or hospital admissions occurring during the observation period using ICD-9-CM codes of GBS: 357.0.

### Definition of exposures

The exposures of interest were the first or second dose of BNT162b2, mRNA-1273, ChAdOx1-S and Ad26.COV2-S vaccines.

The exposure risk interval was defined as [0–42) days after first or second dose administration (vaccination date), which included day 0, the day of vaccination, according to Brighton Collaboration guidance [25]. The unexposed baseline interval (reference period) was defined as any time of observation out of the risk intervals (before, between or after the risk intervals). According to the vaccination schedules of BNT162b2 (21-day interval between the first and second dose) and mRNA-1273 vaccines (28-day interval between the first and second dose), the 42-day risk intervals overlap and, consequently, the risk interval after first dose may end after the second dose. The convention in the SCCS methodology is that the most recent exposure period takes precedence over a previous exposure, and the parameterization of the SCCS model is adjusted accordingly.

### Statistical analysis

Characteristics of the cohort of vaccinated persons and GBS cases were described by age, sex, comorbidities and co-medications.

For each study vaccine, the SCCS model was fitted using unbiased estimating equations to estimate the RI and their 95% Confidence Intervals (95% CI). To handle event-dependent exposures, the SCCS model was properly modified considering a counterfactual exposure history for any exposures arising after occurrence of an event [19–24]. Six 45-day calendar periods were considered as time-varying covariate controlling for the seasonal effect. We also estimated the Excess of Cases (EC) per 100,000 vaccinated. We first calculated the number of EC due to the vaccine in the risk period from the attributable fraction formula *A*F = [(RI−1)÷RI] multiplied by the number of events in that risk period. Then the EC per 100,000 vaccinated were obtained by dividing number of EC due to the vaccine by the number of vaccinated per 100,000.

The 95% CIs were estimated by non-parametric bootstrapping methodology (10,000 replications) [26]. We carried out subgroup analyses by age group (12–39, 40–59, ≥60 years), sex and vaccine product (BNT162b2, mRNA-1273, ChAdOx1-S and Ad26.COV2-S). We performed several sensitivity analyses to assess the robustness of the results. First, we explored the seasonal effect by removing the calendar time factor; second, we investigated the effect of the SARS-CoV-2 infection by restricting the analyses to subjects without a positive SARS-CoV-2 test during the study period; third, we explored the assumption that the most recent exposure period takes precedence over a previous exposure, fitting a common parameter for both doses. Moreover, to support the choice of the modified SCCS model, other sensitivity analyses were conducted using the standard SCCS method: a) beginning observation at time 0; b) beginning observation at exposure (starting the observation time at the first and second dose); c) including a [–28–0) day pre-risk period. The analyses were performed using R version 4.2.2 (R Core Team 2021) with SCCS package [27] and STATA version 16.1.

### Ethics and permissions

This study was approved by the National Unique Ethics Committee for the evaluation of clinical trials of medicines for human use and medical devices for patients with COVID-19 of the National Institute for Infectious Diseases “Lazzaro Spallanzani” in Rome (ordinance n. 335, 17/05/2021 and n. 399, 02/09/2021). The informed consent could not be obtained since this retrospective study used exclusively data which are routinely gathered by the Italian Regions to inform policy decisions and more effective public services and included in large regional health care registry. Ethics Committee waived the requirement for informed consent.

## Results

Between 27 December 2020 and 30 September 2022, the COVID-19 vaccination registries of the five participating Italian Regions included a total of 27,889,821 administered doses. Among them, 179,926 (0.65%) doses were excluded for missing or inconsistent information, and 670,969 (2.41%) doses were excluded for other reasons (exclusion of dose 3 or out of the observation period) (Fig 1).

**Fig 2:**
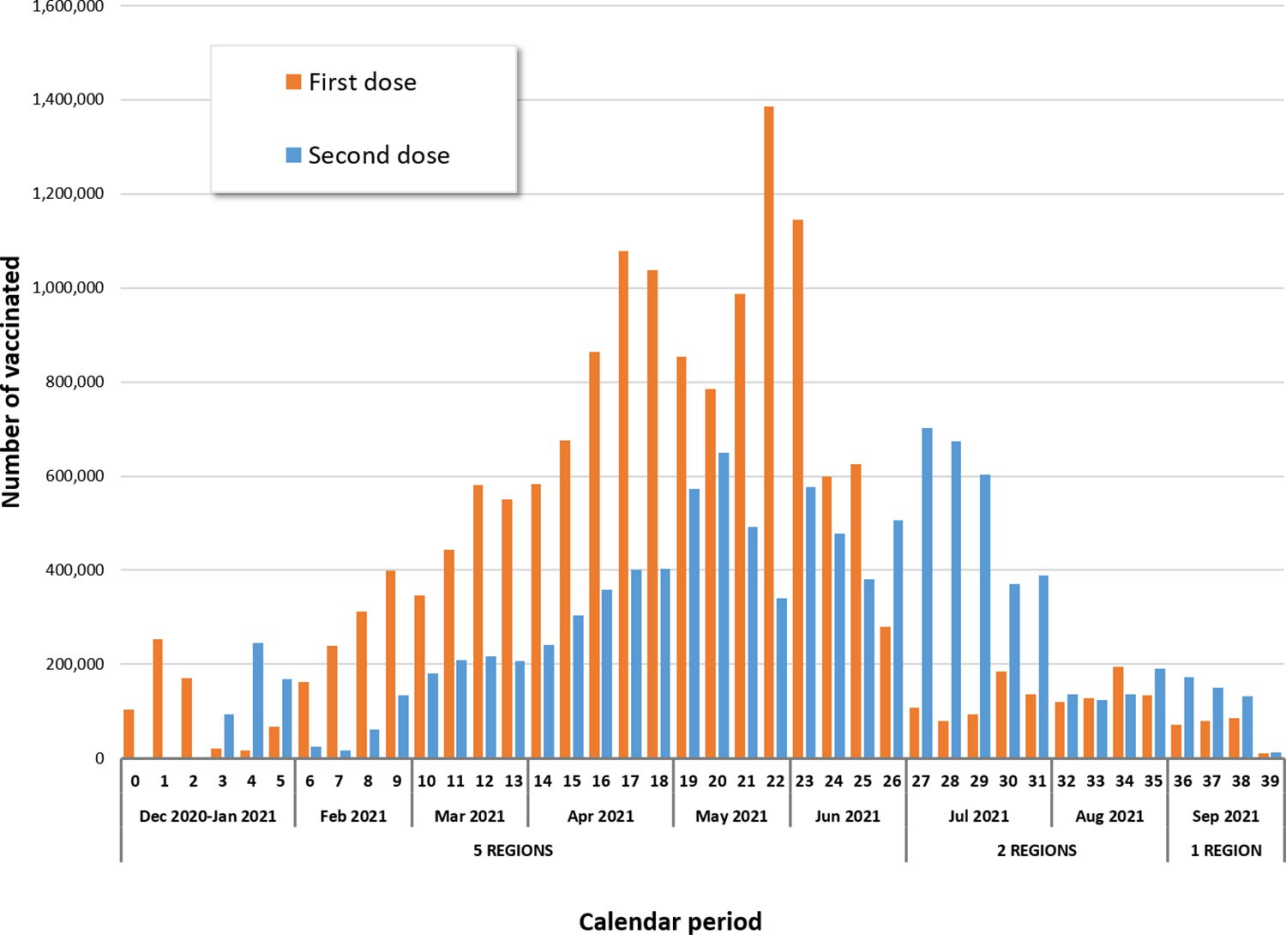
Distribution of first and second dose vaccine administrations by calendar week (27 December 2020–30 September 2021).

Finally, our study population included 15,986,009 persons aged 12 years or over receiving 27,038,926 first or second dose of anti-COVID-19 vaccines (Figs 1, 2 and Table 1), with a median follow-up time of 270 days (interquartile range 237–270 days). The overall vaccination coverage (VC) in the study population, was 67.6% and exceeded 85% in persons ≥60 years of age. The VC by Region was: Lombardia (85.6%), Friuli Venezia Giulia (71.3%), Veneto (56.0%), Emilia Romagna (56.3%) and Lazio (54.3%). Depending on the vaccination registry update and, consequently, on the length of the observation period, we observed the highest VCs in Lombardia and Friuli Venezia Giulia.

**Table 1:** Characteristics of vaccinated population by anti-COVID-19 vaccine brand (27 December 2020–30 September 2021).

Vaccine brand	N.	N. doses	Median age	<40 years	40–59 years	≥60 years	M/F ratio
	vaccinates		[IQ range]				
BNT162b2	10,833,284	18,899,505	53 [40–69]	2,669,912	4,177,622	3,985,750	0.91
mRNA-1273	1,706,979	3,037,506	51 [36–69]	501,782	609,086	596,111	0.94
ChAdOx1-S	2,863,950	4,520,119	64 [54–72]	280,077	677,866	1,906,007	0.85
Ad26.COV2-S	581,796	581,796	57 [51–64]	39,276	307,937	234,583	1.18
**Total**	**15,986,009**	**27,038,926**	**56 [42–70]**	**3,491,047**	**5,772,511**	**6,722,451**	**0.91**

N.: number; IQ: interquartile; M/F: males/ females

Dates of regional vaccination registry update: Veneto, Emilia Romagna, Lazio (Dec. 2020-Jun. 2021); Friuli Venezia Giulia (Dec. 2020-Aug. 2021); Lombardia (Dec. 2020-Sep. 2021).

During the anti-COVID-19 vaccination campaign, in the five study Regions, four different vaccines were used, two mRNA vaccines (BN162b2: n = 10,833,284, 67.8%; mRNA-1273: n = 1,706,979, 10.7%) and two viral vector vaccines (ChAdOx1-S: n = 2,863,950, 17.9%; Ad26.COV2-S: n = 581,796, 3.6%) (Table 1). The 15,986,009 vaccinated subjects had a median age of 56 years, interquartile range (IQR) [42–70] and 52% were females. The clinical characteristics of the vaccinated subjects obtained from data available in the archives of pharmaceutical prescriptions, hospital admissions and exemptions are shown in S1 Table.

In Table 2 are shown the characteristics of the GBS cases. During the study period, among 15,986,009 vaccinees,287 had a new diagnosis of GBS (median age 65 years, IQR [54–76]), 184 cases (64.1%) after the first dose of anti-COVID-19 vaccine. The incidence was 2.60 per 100.000 person-years. Among 10,833,284 recipients of BNT162b2 vaccine, 187 had GBS. Among 1,706,979 recipients of mRNA-1273, 25 individuals had GBS. Among 2,863,950 ChAdOx1-S vaccinees and 581,796 Ad26.COV2-S vaccinees there were 58 and 17 new cases of GBS. Thirteen deaths for all causes of death were observed during the study observation period, with a median age of 80 years, IQR [74–81] and 54% with a Charlson Index greater than 1.

**Table 2:** Characteristics of GBS cases by vaccine brand, n. 287 (27 December 2020–30 September 2021).

	Total (n. 287)	BNT162b2 (n. 187)	mRNA-1273 (n. 25)	ChAdOx1-S (n. 58)	Ad26.COV2-S (n. 17)
**Median age, yrs [IQR]**	65 [54–76]	65 [52–78]	64 [54–78]	68 [59–72]	63 [55–68]
**Age, n (%)**					
<40 yrs	26 (9.1)	25 (13.4)	0	0	1 (5.9)
40–59 yrs	76 (26.5)	47 (25.1)	10 (40.0)	15 (25.9)	4 (23.5)
≥60 yrs	185 (64.5)	115 (61.5)	15 (60.0)	43 (74.1)	12 (70.6)
**Sex, n. (%)**					
Males	171 (59.6)	113 (60.4)	16 (64.0)	32 (55.2)	10 (58.8)
Females	116 (40.4)	74 (39.6)	9 (36.0)	26 (44.8)	7 (41.2)
**Regions, n. (%)**					
Lombardia	144 (50.2)	99 (52.9)	13 (52.0)	26 (44.8)	6 (35.3)
Friuli Venezia Giulia	20 (7.0)	10 (5.3)	3 (12.0)	6 (10.3)	1 (5.9)
Veneto	31 (10.8)	22 (11.8)	1 (4.0)	6 (10.3)	2 (11.8)
Emilia Romagna	50 (17.4)	30 (16.0)	7 (28.0)	7 (12.1)	6 (35.3)
Lazio	42 (14.6)	26 (13.9)	1 (4.0)	13 (22.4)	2 (11.8)
n. prescriptions* **median [IQR]**	12 [3–28]	15 [5–31]	16 [5–39]	8 [1–23]	3 [0–6]
**Hospitalizations≥1** ** **, n. (%)**	135 (47.0)	112 (59.9)	12 (48.0)	7 (12.1)	4 (23.5)
**Charlson index≥1** *** **, n. (%)**	134 (46.7)	95 (50.8)	18 (72.0)	18 (31.0)	3 (17.6)
**Comorbidities, n. (%)**					
Hypertension	163 (56.8)	110 (58.8)	17 (68.0)	30 (51.7)	6 (35.3)
Ulcer disease	108 (37.6)	87 (46.5)	8 (32.0)	11 (19.0)	2 (11.8)
Infection	103 (35.9)	77 (41.2)	8 (32.0)	15 (25.9)	3 (17.6)
Cardiovascular/cerebrovascular diseases	95 (33.1)	73 (39.0)	9 (36.0)	10 (17.2)	3 (17.6)
Hematologic disease	86 (30.0)	67 (35.8)	9 (36.0)	8 (13.8)	2 (11.8)
Neurological diseases (excl. Dementia)	80 (27.9)	60 (32.1)	12 (48.0)	8 (13.8)	0
Neoplasms	50 (17.4)	37 (19.8)	5 (20.0)	6 (10.3)	2 (11.8)
Diabetes mellitus	50 (17.4)	36 (19.3)	6 (24.0)	8 (13.8)	0
Acute and other chronic pulmonary disease	49 (17.1)	37 (19.8)	7 (28.0)	2 (3.4)	3 (17.6)
COPD	30 (10.5)	17 (9.1)	8 (32.0)	5 (8.6)	0
Chronic kidney failure	10 (3.5)	7 (3.7)	3 (12.0)	0	0
Rheumatic diseases	7 (2.4)	6 (3.2)	1 (4.0)	0	0
Hepatopathy	6 (2.1)	2 (1.1)	4 (16.0)	0	0
Regional and ulcerative colitis	3 (1.0)	3 (1.6)	0	0	0
Dementia	2 (0.7)	2 (1.1)	0	0	0
HIV	1 (0.3)	0	1 (4.0)	0	0
**Comedications, n. (%)**					
Corticosteroids for systemic use	65 (22.6)	47 (25.1)	7 (28.0)	6 (10.3)	5 (29.4)
NSAID use	29 (10.1)	21 (11.2)	1 (4.0)	5 (8.6)	2 (11.8)
**Deaths, n. (%)**	13 (4.5)	10 (5.3)	2 (8.0)	1 (1.7)	0
**n. events ≥ first dose, n. (%)**	184 (64.1)	103 (55.1)	15 (60.0)	55 (94.8)	11 (64.7)
**n. positive SARS-CoV-2 test, n. (%)**	33 (11.5)	21 (11.2)	5 (20.0)	3 (5.2)	4 (23.5)

*all drug prescriptions within the last 12 months prior to anti-COVID-19 first dose administration

** any hospital admissions within the 2 years prior to anti-COVID-19 first dose administration

*** within the 5 years prior to anti-COVID-19 first dose administration

Yrs: years; IQR: Interquartile Range; n.: number excl.: excluding; COPD: Chronic Obstructive Pulmonary Disease; HIV: Human Immunodeficiency Virus; NSAID: Non-Steroidal Anti-Inflammatory Drugs

The number of GBS cases in the 42-day risk interval was 67 after the first dose and 41 after the second dose (Table 3). In the 42-day risk interval, increased risks were observed after the administration of first dose (RI = 6.83; 95% CI 2.14–21.85) and second dose (RI = 7.41; 95% CI 2.35–23.38) for mRNA-1273 vaccine, corresponding to estimated 0.4 (95% CI 0.1–0.7) and 0.3 (95% CI 0.05–0.6) EC per 100,000 vaccinated respectively. Considering the overlapping of the 42-day risk periods between the first and second dose for mRNA-1273 vaccine, it cannot be excluded that the increased risk observed in the 42-day risk period after the second dose might be partially driven by the effect of the first dose. Increased risk was also observed after the first dose of ChAdOx1-S vaccine (RI = 6.52; 95% CI 2.88–14.77), corresponding to estimated 1.0 (95% CI 0.7–1.3) EC per 100,000 vaccinated. We did not observe evidence of an increased risk of GBS after vaccination with BNT162b2 and Ad26.COV2-S vaccines (Table 3 and S2 Table).

**Table 3:** Relative Incidences estimated by Self-Controlled Case Series model by vaccine brand and dose: 287 Guillain Barré Syndrome events in the anti-COVID-19 vaccinated population (27 December 2020–30 September 2021).

	Dose	Risk period	BNT162b		mRNA-1273^		ChAdOx1-S		Ad26.COV2-S	
			Events (n. 187)	RI (95% CI)	Events (n. 25)	RI (95% CI)	Events (n. 58)	RI (95% CI)	Events (n. 17)	RI (95% CI)
**Total**		*Ref*.	*138*	*1*	*13*	*1*	*18*	*1*	*10*	*1*
	1	[0–42)	19	0.85 (0.49–1.48)	7	6.83 (2.14–21.85)	34	6.52 (2.88–14.77)	7	1.94 (0.32–11.69)
	2	[0–42)	30	1.30 (0.80–2.10)	5	7.41 (2.35–23.38)	6	3.56 (0.31–40.29)		
**Males**		*Ref*.	*89*	*1*	*8*	*1*	*11*	*1*	*6*	*1*
	1	[0–42)	9	0.64 (0.30–1.36)	3	5.26 (0.94–29.42)	16	4.94 (1.84–13.28)	4	1.06 (0.15–7.39)
	2	[0–42)	15	1.06 (0.56–2.00)	5	16.50 (3.01–90.56)	5	1.54 (0.13–18.39)		
**Females**		*Ref*.	*49*	*1*	*5*	*1*	*7*	*1*	*4*	*1*
	1	[0–42)	10	1.19 (0.55–2.55)	4	13.44 (2.83–63.80)	18	7.14 (1.94–26.19)	3	2.35 (0.18–30.00)
	2	[0–42)	15	1.84 (0.85–4.00)	0	-	1	*		
**12–39 yrs**		*Ref*.	*18*	*1*	*0*	*1*	*0*	*1*	*1*	*1*
	1	[0–42)	4	0.68 (0.15–3.14)	0	-	0	-	0	-
	2	[0–42)	3	0.64 (0.13–3.29)	0	-	0	-		
**40–59 yrs**		*Ref*.	*34*	*1*	*6*	*1*	*4*	*1*	*2*	*1*
	1	[0–42)	6	1.02 (0.36–2.84)	3	*	10	4.50 (1.37–14.79)	2	*
	2	[0–42)	7	1.63 (0.61–4.37)	1	*	1	*		
**≥60 yrs**		*Ref*.	*86*	*1*	*7*	*1*	*14*	*1*	*7*	*1*
	1	[0–42)	9	0.74 (0.35–1.57)	4	8.03 (2.08–31.03)	24	6.84 (2.56–18.28)	5	1.78 (0.27–11.58)
	2	[0–42)	20	1.21 (0.65–2.25)	4	7.71 (2.38–24.97)	5	1.97 (0.13–30.39)		

RI: Relative Incidence; CI: Confidence Interval; n.: number; Ref.: reference; yrs: years

^3 calendar periods of 90 days

* RI not estimable by the SCCS model

### Subgroup and sensitivity analyses

In the subgroup analysis by sex (Table 3), an increased risk of GBS was observed in the 0–42 days risk period among both males and females after mRNA-1273 vaccine. More specifically, in males a marginally non-statistically significant increased risk was observed after the first dose, (RI = 5.26; 95% CI 0.94–29.42; p = 0.06) and the second dose (RI = 16.50; 95% CI 3.01–90.56); in females the increased risk was confined to the first dose (RI = 13.44; 95% CI 2.83–63.80). There was also evidence of an increased risk after a first dose of ChAdOx1-S in males (RI = 4.94; 95% CI 1.84–13.28) and females (RI = 7.14; 95% CI 1.94–26.19).

In the subgroup analysis by age (Table 3), there was evidence of an increased risk of GBS with mRNA-1273 vaccine among those aged ≥60 years after the first (RI = 8.03; 95% CI 2.08–31.03) and second dose (RI = 7.71; 95% CI 2.38–24.97). After a first dose of ChAdOx1-S there was evidence of an increased risk of GBS in those aged 40–59 (RI = 4.50; 95% CI 1.37–14.79) and in those aged ≥60 years (RI = 6.84; 95% CI 2.56–18.28). In the subgroup analysis by age and sex, evidence of an increased risk of GBS after vaccination with BNT162b2 and Ad26.COV2-S vaccines were not observed.

Results of the sensitivity analyses which were performed to assess the robustness of the SCCS methodology, were similar with the results of the main analysis (Table 4).

**Table 4:** Sensitivity analyses: Relative Incidences estimated by Self-Controlled Case Series model by vaccine brand and dose: 287 Guillain Barré Syndrome events in the anti-COVID-19 vaccinated population (27 December 2020–30 September 2021).

	Dose	Risk period	RI (95% CI)			
			BNT162b	mRNA-1273	ChAdOx1-S	Ad26.COV2-S
**Modified SCCS**						
** without seasonal effect**		*Ref*.	*1*	*1*	*1*	*1*
	1	[0–42)	0.80 (0.47–1.38)	4.10 (1.35–12.42)	4.74 (2.48–9.06)	3.24 (0.87–12.05)
	2	[0–42)	1.23 (0.77–1.95)	3.97 (1.03–15.30)	6.22 (0.85–45.55)	
without positive SARS-CoV-2 test*		*Ref*.	*1*	*1*		
	1	[0–42)	0.76 (0.41–1.43)	8.84 (2.94–26.58)	6.83 (2.88–16.22)	1.40 (0.19–10.29)
	2	[0–42)	1.33 (0.81–2.19)	8.52 (2.84–25.53)	3.63 (0.32–41.38)	
** combining doses**		*Ref*.	*1*	*1*	*1*	
	1+2	[0–42)	0.99 (0.63–1.54)	7.01 (2.54–19.31)	6.20 (2.78–13.87)	
**Standard SCCS**						
** beginning observation at the time 0**		*Ref*.	*1*	*1*	*1*	
	1	[0–42)	0.91 (0.60–1.40)	5.28 (1.98–14.07)	9.54 (4.38–20.76)	2.38 (0.70–8.04)
	2	[0–42)	1.80 (1.15–2.82)	3.93 (1.14–13.49)	4.17 (1.30–13.41)	
beginning observation at exposure**		*Ref*.	*1*	*1*	*1*	*1*
	1	[0–42)	0.74 (0.46–1.17)	4.21 (1.40–12.73)	4.64 (2.48–8.70)	3.24 (0.91–11.58)
	2	[0–42)	1.17 (0.71–1.92)	3.97 (0.94–16.78)	6.22 (0.74–52.00)	
** including a [-28,0) pre-risk period**		*Ref*.	1	1	1	1
	1	[0–42)	0.80 (0.52–1.23)	4.57 (1.72–12.13)	7.39 (3.32–16.43)	1.90 (0.48–7.55)
	2	[0–42)	1.60 (1.02–2.51)	3.56 (1.04–12.20)	3.98 (1.25–12.73)	

RI: Relative Incidence; CI: Confidence Interval; SCCS: Self-Controlled Case Series; Ref.: reference

*Analyses restricted to 254 GBS cases without a positive SARS-CoV-2 test during the study period (166 with BNT162b; 20 with mRNA-1273; 55 with ChAdOx1-S; 13 with Ad26.COV2-S)

**Analyses restricted to 184 GBS cases following first dose (103 with BNT162b; 15 with mRNA-1273; 55 with ChAdOx1-S; 11 with Ad26.COV2-S) and 92 GBS cases following second dose (77 with BNT162b; 8 with mRNA-1273; 7 with ChAdOx1-S)

## Discussion

This large SCCS study, covering about 16 million people, found evidence of an increased risk of GBS after administration of first and second dose of mRNA-1273 and first dose of ChAdOx1-S in the risk period of 0–42 days. We did not observe evidence of an increased risk of GBS following first and second dose of BNT162b nor after Ad26.COV2-S vaccination. Assuming a causal effect, the number of the estimated EC were low with 0.4 and 0.3 EC per 100,000 vaccinated for first and second dose of mRNA-1273 and 1.0 EC per 100,000 vaccinated for the first dose of ChAdOx1-S.

Subgroup analyses by sex found an increased risk of GBS with mRNA-1273 after the second dose in males (with results of a borderline statistical significance after the first dose) and after the first dose in females. An increased risk was also observed after the first dose of ChAdOx1-S both in males and females. Stratifying data by age, an increased risk was observed in those aged 40–59 years after first dose of ChAdOx1-S. Additionally, we found evidence of an association with GBS of both mRNA-1273 and ChAdOx1-S vaccines in those aged ≥60 years.

Our results are in line with previous findings from surveillance studies in England with SCCS study design [9, 10] investigating the association between ChAdOx1-S and BNT162b vaccines with acute neurological outcomes. For example, Walker *et al*. [10], that used the standard SCCS method and not the adapted to event-dependent exposure, found an increased incidence of GBS after a first dose of ChAdOx1-S at 4–42 days post vaccination (Incidence Rate Ratio-IRR = 2.85; 95% CI 2.33–3.47) and no evidence of an association after first and second dose for BNT162b. This study did not investigate the relationship between mRNA-1273 and GBS due to limited power. Similarly, the study of Patone *et al*. [9] (using the standard SCCS method and not the event dependent exposure), observed an increased risk of GBS following first dose of ChAdOx1-S at 1–28 days after vaccination (IRR = 2.04; 95% CI 1.60–2.60) but not after first dose of BNT162b vaccination. This study had insufficient length of follow-up to assess the effect of the second dose of COVID-19 vaccines.

Our study has the strengths of combining a larger study population and a longer follow-up than previous studies which permitted us to obtain precise estimates with the SCCS model for the first and second dose of mRNA-1273 and the first dose of Ad26.COV2-S, including sex and age-specific estimates. To date, to our knowledge, there are no studies that provide such information. Our findings, therefore, may be relevant for the periodic update of the benefit-risk assessment and for developing sex and age-specific recommendations on COVID-19 vaccination.

Furthermore, to ensure representativeness of the Italian population and accuracy and completeness data on COVID-19 vaccination and outcome, we were able to access official data from five large and linked regional databases of high quality.

Another advantage of our study is inherently to the SCCS method which is not susceptible to confounding by known and unknown factors that are time-invariant during the study period. The seasonality effect was also included in the models as an important time variant confounding factor. A further strength is the application of the SCCS method modified to handle event-dependent exposure as already described in our recent SCCS study on the association of COVID-19 mRNA vaccines and myocarditis/pericarditis [12].

This approach, specifically for COVID-19 vaccines, has been demonstrated by Ghebremichael-Weldeselassie et colleagues to correct the overestimation of RIs which tends to occur when vaccination is deferred for long periods or even indefinitely after an event, which was the case of our study [19]. The authors noted that one of the alternative formulations of the SCCS model, i.e. the standard model where a pre-vaccination risk period is included, provides valid estimates only when the event led to a brief delay in vaccination.

When applying the SCCS method modified to avoid biased RI estimates, the authors also indicated the importance to include unvaccinated cases or at least a certain proportion of vaccinated cases for whom the event occurs before the first vaccine dose [19]. In our study this proportion was 36%, therefore there is a potential for a moderate inflation of the RIs. In fact, Ghebremichael-Weldeselassie et colleagues found an overestimation of RI equal to 10% with inclusion of a proportion of 50%.

Nevertheless, we carried out several sensitivity analyses to investigate the robustness of our results to the assumptions of the SCCS model which revealed similar results to the main analysis (Table 4). Notably, the sensitivity analysis using the standard SCCS method with the observation time starting at first or second vaccination provides greater confidence in the reliability of the RI estimates.

This study has limitations that deserve comments. First, since we did not consider outpatient data, GBS cases with mild symptoms may have been not captured, with potential for underestimation of the number of the events, although is reassuring that our estimate of 2.6 per 100.000 person-years is similar with the estimate of GBS of an Italian previous study based on validated clinical records [28]. Second, although our sample included more than 15 million of vaccinated, we could not obtain precise estimates in some subgroup analyses, considering the rarity of the event. Third, GBS cases were not validated through review of medical records, therefore we cannot exclude potential misclassification. However, information bias is less likely to have occurred since, by design, data on the exposures, outcome and covariates were collected irrespective of the research question.

## Conclusions

In conclusion, our population-based study, found an increased risk of GBS after administration of first and second dose of mRNA-1273 and first dose of ChAdOx1-S, however these risks resulted in a small number of excess cases. Age-specific estimates indicated an increased risk in those aged 40–59 years and aged ≥60 years vaccinated with ChAdOx1-S. Additionally, an increased risk of GBS was found with mRNA-1273 in those aged ≥60 years. No evidence of an increased risk of GBS was observed following each of BNT162b and Ad26.COV2-S vaccine dose. Further and larger analytical studies are needed to confirm our results.

With the implementation of a worldwide COVID-19 vaccination campaign it is important the continuous monitoring of the suspected adverse events of these new vaccines as key component of any vaccination program for the evaluation of benefit-risk profile of vaccination.

These findings are likely to be of relevance to regulators, health professionals and developers of clinical guidelines in the risk benefit evaluations of the COVID-19 vaccines.

## Supporting information

S1 Checklist RECORD PE checklist of items. (PDF)

S1 Table Characteristics of vaccinated population aged ≥12 years by age group (27 December 2020–30 September 2021). (PDF)

S2 Table Excess of cases estimates with 95% CIs for GBS cases corresponding to exposures with RIs estimated in the 42 days risk period with p<0.05. (PDF)
