# Supplementary material for: Post-marketing active surveillance of Guillain Barré Syndrome following COVID-19 vaccination in persons aged ≥12 years in Italy: A multi-database self-controlled case series study
Source: PLoS One. 2024 Jan 19;19(1):e0290879. doi: 10.1371/journal.pone.0290879 (PMC10798452; doi:10.1371/journal.pone.0290879)
Supplement: S1 Table — (PDF) [file pone.0290879.s002.pdf]

SUPPORTING INFORMATION

Post-marketing active surveillance of Guillain Barré Syndrome following COVID-19 vaccination in persons aged ≥12 years in Italy: a multi-database self-controlled case series study

Morciano C & Spila Alegiani S, et al.

S1 Table. Characteristics of vaccinated population aged ≥12 years by age group (27 Dec. 2020-30 Sep. 2021)

	Total	Age 12-39 years	Age 40-59 years	Age ≥60 years
	N. (%)	N. (%)	N. (%)	N. (%)
**Number of subjects**	15,986,009	3,491,047	5,772,511	6,722,451
**Sex, n. (%)**				
Males	7,607,597 (47.6)	1,750,701 (50.1)	2,830,813 (49.0)	3,026,083 (45.0)
Females	8,378,412 (52.4)	1,740,346 (49.9)	2,941,698 (51.0)	3,696,368 (55.0)
**Regions, n. (%)**				
Lombardia	7,683,100 (48.1)	2,356,666 (67.5)	2,664,257 (46.2)	2,662,177 (39.6)
Friuli Venezia Giulia	780,904 (4.9)	190,668 (5.5)	256,566 (4.4)	33,3670 (5.0)
Veneto	2,457,271 (15.4)	260,949 (7.5)	949,680 (16.5)	1,246,642 (18.5)
Emilia Romagna	2,257,835 (14.1)	360,267 (10.3)	817,441 (14.2)	1,08,0127 (16.1)
Lazio	2,806,899 (17.6)	322,497 (9.2)	1,084,567 (18.8)	1,399,835 (20.8)
**n. prescriptions* median [IQR]**	3 [0-16]	0 [0-1]	1 [0-7]	16 [5-32]
**Hospitalizations≥1**, n. (%)**	2,328,902 (14.6)	364,043 (10.4)	612,087 (10.6)	1,352,772 (20.1)
**Charlson index≥1***, n. (%)**	1,492,389 (9.3)	46,029 (1.3)	249,828 (4.3)	1,196,532 (17.8)
**Comorbidities, n. (%)**				
Hypertension	5,544,989 (34.7)	60,820 (1.7)	1,124,452 (19.5)	4,359,717 (64.9)
Infection	4,119,897 (25.8)	650,170 (18.6)	1,350,769 (23.4)	2,118,958 (31.5)
Ulcer disease	3,400,860 (21.3)	154,697 (4.4)	770,887 (13.4)	2,475,276 (36.8)
Cardiovascular/cerebrovascular diseases	2,782,479 (17.4)	67,212 (1.9)	364,056 (6.3)	2,351,211 (35.0)
Hematologic disease	2,464,774 (15.4)	183,137 (5.2)	504,280 (8.7)	1,777,357 (26.4)
Neurological diseases (excl. Dementia)	1,932,003 (12.1)	147,406 (4.2)	519,760 (9.0)	1,264,837 (18.8)
Neoplasms	1,569,419 (9.8)	46,242 (1.3)	330,143 (5.7)	1,193,034 (17.7)
Diabetes mellitus	1,407,622 (8.8)	54,846 (1.6)	268,801 (4.7)	1,083,975 (16.1)
COPD	1,273,224 (8.0)	201,918 (5.8)	362,903 (6.3)	708,403 (10.5)
Acute and other chronic pulmonary disease	395,814 (2.5)	12,792 (0.4)	61,130 (1.1)	321,892 (4.8)
Rheumatic diseases	324,731 (2.0)	31,803 (0.9)	106,698 (1.8)	186,230 (2.8)
Chronic kidney failure	238,511 (1.5)	7,223 (0.2)	33,040 (0.6)	198,248 (2.9)
Hepatopathy	221,532 (1.4)	12,153 (0.3)	81,508 (1.4)	127,871 (1.9)
Dementia	124,112 (0.8)	88 (0.003)	1,469 (0.03)	122,555 (1.8)
HIV	65,754 (0.4)	6,832 (0.2)	34,509 (0.6)	24,413 (0.4)
Regional and ulcerative colitis	82,109 (0.5)	13,861 (0.4)	32,522 (0.6)	35,726 (0.5)
Cystic fibrosis	2,067 (0.01)	1,359 (0.04)	546 (0.01)	162 (0.002)
**Comedications, n. (%)**				
Corticosteroids for systemic use	1,346,138 (8.4)	130,966 (3.8)	460,120 (8.0)	755,052 (11.2)
NSAID use	917,928 (5.7)	34,358 (1.0)	270,876 (4.7)	612,694 (9.1)
Estroprogestinics use	186,545 (1.2)	34,020 (1.0)	96,349 (1.7)	56,176 (0.8)
**Deaths (all causes), n. (%)**	77,808 (0.5)	303 (0.01)	2,967 (0.1)	74,538 (1.1)

all drug prescriptions within the last 12 months prior to anti-COVID-19 first dose administration; ** any hospital admissions within the 2 years prior to anti-COVID-19 first dose administration; * within the 5 years prior to anti-COVID-19 first dose administration*

n.: number; IQR: Interquartile Range; excl.: excluding; COPD: Chronic Obstructive Pulmonary Disease; HIV: Human Immunodeficiency Virus; NSAID: Non-Steroidal Anti-Inflammatory Drugs
