# Supplementary material for: Post-marketing active surveillance of Guillain Barré Syndrome following COVID-19 vaccination in persons aged ≥12 years in Italy: A multi-database self-controlled case series study
Source: PLoS One. 2024 Jan 19;19(1):e0290879. doi: 10.1371/journal.pone.0290879 (PMC10798452; doi:10.1371/journal.pone.0290879)
Supplement: S2 Table — (PDF) [file pone.0290879.s003.pdf]

SUPPORTING INFORMATION

Post-marketing active surveillance of Guillain Barré Syndrome following COVID-19 vaccination in persons aged ≥12 years in Italy: a multi-database self-controlled case series study

Morciano C & Spila Alegiani S, et al.

S2 Table. Excess of cases estimates with 95% CIs for GBS cases corresponding to exposures with RIs estimated in the 42 days risk period with p<0.05

Vaccine exposure	Cases in 42 days risk period	Relative Incidence	Attributable Fraction	Excess cases due to the vaccine	Doses	EC x 100,000 vaccinated (95% CI)
**Dose 1 mRNA-1273**	7	6.83	0.85	6.0	1,706,979	0.4 (0.1-0.7)
**Dose 2 mRNA-1273**	5	7.41	0.87	4.3	1,330,527	0.3 (0.05-0.6)
**Dose 1 ChAdOx1-S**	34	6.52	0.85	28.8	2,863,950	1.0 (0.7-1.3)

EC: Excess of cases; CI: Confidence interval
